# The Contribution of Gut Barrier Changes to Multiple Sclerosis Pathophysiology

**DOI:** 10.3389/fimmu.2019.01916

**Published:** 2019-08-28

**Authors:** Maria Chiara Buscarinu, Arianna Fornasiero, Silvia Romano, Michela Ferraldeschi, Rosella Mechelli, Roberta Reniè, Emanuele Morena, Carmela Romano, Giulia Pellicciari, Anna Chiara Landi, Marco Salvetti, Giovanni Ristori

**Affiliations:** ^1^Department of Neurosciences, Mental Health and Sensory Organs, Faculty of Medicine and Psychology, Centre for Experimental Neurological Therapies, Sapienza University, Rome, Italy; ^2^Department of Neurology and Psychiatry, Sapienza University, Rome, Italy; ^3^Department of Human Science and Promotion of Quality of Life, San Raffaele Roma Open University, Rome, Italy; ^4^IRCCS Istituto Neurologico Mediterraneo (INM) Neuromed, Pozzilli, Italy

**Keywords:** gut barrier, intestinal permeability, microbiota, multiple sclerosis, neuro-inflammatory diseases

## Abstract

The gut barrier consists of several components, including the mucus layer, made of mucins and anti-bacterial molecule, the epithelial cells, connected by tight junction proteins, and a mixed population of cells involved in the interplay with microbes, such as M cells, elongations of “antigen presenting cells” dwelling the lamina propria, intraepithelial lymphocytes and Paneth cells secreting anti-bacterial peptides. Recently, the influence of intestinal permeability (IP) changes on organs far from gut has been investigated, and IP changes in multiple sclerosis (MS) have been described. A related topic is the microbiota dysfunction that underpins the development of neuroinflammation in animal models and human diseases, including MS. It becomes now of interest to better understand the mechanisms through which IP changes contribute to pathophysiology of neuroinflammation. The following aspects seem of relevance: studies on other biomarkers of IP alterations; the relationship with known risk factors for MS development, such as vitamin D deficiency; the link between blood brain barrier and gut barrier breakdown; the effects of IP increase on microbial translocation and microglial activation; the parallel patterns of IP and neuroimmune changes in MS and neuropsychiatric disorders, that afflict a sizable proportion of patients with MS. We will also discuss the therapeutic implications of IP changes, considering the impact of MS-modifying therapies on gut barrier, as well as potential approaches to enhance or protect IP homeostasis.

## Introduction

The requirement for different functions is reflected by the structural complexity of the intestinal surface. Its role as a barrier relies on three components. The layer of mucus contributes to separate the microbiota from the upper part of the epithelium. The epithelial cells, with tight junction (TJ), regulate the paracellular permeability. A third component with immunological functions includes M cells and elongations of “antigen presenting cells” dwelling the lamina propria, that scan the luminal antigens, intraepithelial lymphocytes, and Paneth cells secreting anti-bacterial peptides. The passage of substances through this physical barrier is possible thanks to trans-cellular or para-cellular transport mechanisms. The first is closely related to the presence of selective transporters, the second is under the control of the proteins that make up the TJ, especially occludin and claudins. The TJ can be assembled or disassembled according to the different signals coming from the intra- and extra-cellular environment. Dietary factors, microbiota composition, cytokines, enzymes and growth factors can all contribute to modulate TJ (1).

The enteric nervous system, consisting of the ganglia of enteric neurons and glial cells able to release important mediators in repair, cell proliferation, epithelial differentiation and TJ changes (2), regulates the intestinal permeability (IP) and represents a communication pathway between the intestinal microenvironment and the CNS. A recent review emphasized the bottom-up connections, which occur through neuroendocrine tissue, such as enterochromaffin cells, and neuroimmune mechanisms, that often involve the vagus nerve. Even the microbiota plays a pivotal role in the communication between intestine and brain through the production of certain substances, such as short chain fatty acids (SCFA) or tryptophan catabolites, that contribute to the homeostasis of IP (3).

The IP changes (IPC) and the dysbiosis appear as virtually co-occurring events, that trigger a vicious circle leading to pathogenic cascades in gut and far-from-gut tissues. In fact, recent evidences coming from children with beta cell autoimmunity, at risk for type 1 diabetes, showed that both increased intestinal permeability and differences in microbiota composition are contemporarily associated with the pre-pathological condition, being thus early events in the development of autoimmunity (4). However, investigations on IPC are relatively rare in neuroinflammation, especially in the human disease, while studies on dysbiosis are already very numerous, and microbiota alterations were deeply investigated in both experimental autoimmune encephalomyelitis (EAE) and MS. Over the last decade several studies on animal models showed that an immune response to gut microbiota is able to promote cerebral autoimmunity driven by the expansion of pro-inflammatory T cells and autoantibodies, at expenses of regulatory T cells (5–8).

Recent studies carried out in MS patients supported the importance of microbiota alterations in disease pathophysiology. Some authors tried to define metagenomic signatures of microbiomes associated to MS: a group reported higher Firmicutes/Bacteroidetes ratio, increase *Streptococcus* and decreased *Prevotella* strains in patients with active disease (9); others found increased *Akkermansia muciniphila* and *Acinetobacter calcoaceticus*, and reduced *Parabacteroides distasonis* in patients compared to controls (10). The effects of human-derived microbiota on EAE is an interesting approach to evaluate the impact of dysbiosis or commensal bacteria on neuroinflammation. The MS-derived microbiota was capable of inducing or worsening experimental models of disease (10, 11), while *Prevotella histicola*, a human gut-derived commensal bacteria, could suppress EAE (12).

Studies on IPC in MS and EAE are not very numerous. Approximately 20 years ago, studying co-morbidity between Crohn's disease and MS, a first finding of increased IP was reported in a minority of cases with MS (13). The recent momentum of the gut-brain axis role in the pathogenesis of neuroinflammation prompted studies on IP changes in EAE. A work showed that an increased IP preceded EAE development and worsened during disease with disruption of TJ. These changes were associated with unbalance of mucosal immunity (prevalence of pro-inflammatory Th1-Th17 subsets over T regulatory cells). The same work also showed that similar alterations of intestinal barrier occurred in the passive model of EAE after transfer of encephalitogenic T cells (14). Starting from the evidence of the plausible pathogenic role of mucosal-associated invariant T cells in MS (15), we decided to investigate further the role of gut in the neuroinflammation, and designed a pilot study on IPC in patients with relapsing-remitting MS and healthy controls, including MS-discordant twin pairs. We reported that an alteration of IP is a relatively frequent event in MS. Data on twins suggested a genetic influence on the determinants of gut barrier disruption. IPC included a deficit of the active mechanism of absorption from intestinal lumen in patients compared to controls (16).

**Figure 1:**
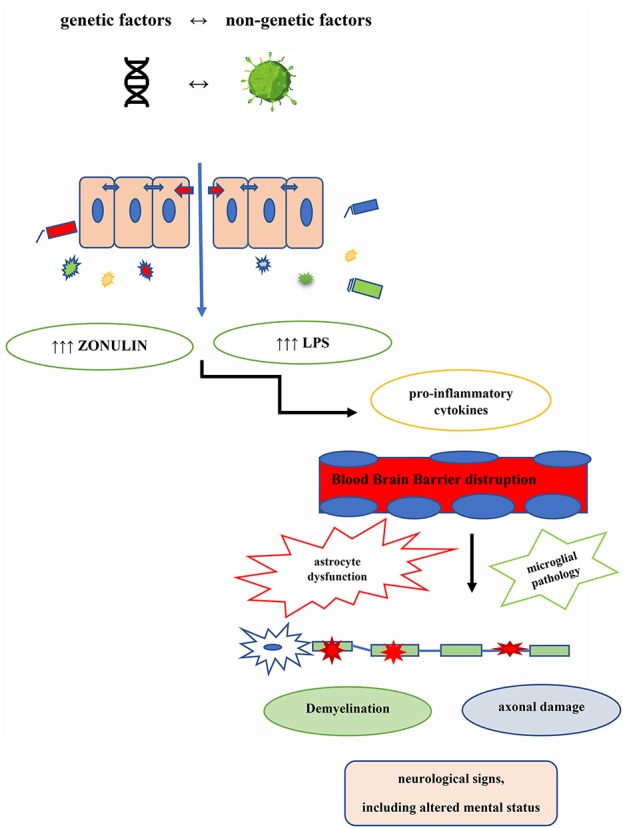
MS pathogenic loop centered on the gut barrier disruption. Genetic and non-genetic aetiologic factors contribute to the co-occurring intestinal permeability changes (IPC) and dysbiosis, that in turn bring about the MS pathogenic cascade.

The topic of IPC in neuroinflammation is currently under active scrutiny, and several lines of investigations are focusing on the plausible relationships between gut barrier disruption and pathophysiologic components of MS (Figure 1), as well as on translational implications based on IPC (Table 1).

**Table 1:** Gut barrier stabilizers or enhancers investigated in chronic inflammation.

**Intervention**	**Disorders with gut barrier disruption**
Larazotide	Celiac disease
Divertin	Experimental inflammatory bowel disease
Food-grade bacteria engineered to produce elafin	Inflammatory bowel disease and gluten-related disorders
Vitamin D	Inflammatory bowel disease and other immune-mediated disorders
Escherichia coli strain Nissle 1917	Experimental autoimmune encephalomyelitis
Approaches targeting the Mincle-Syk axis in gut dendritic cells	Commensals deprivation
Obeticolic acid	Alcoholic hepatitis, non-alcoholic steatohepatitis, and primary biliary cirrhosis
Microbiota transplantation	Clostridium difficile infection

### Intestinal Permeability Changes and MS Pathophysiology

An important issue to be faced for IPC evaluation regards the methodological improvement of IP metrics. Recent approaches, using multi-sugar tests, allow to study different segments of gastro-intestinal tract and may prove to be more informative to understand the level especially involved in MS pathogenesis (17). Among the peripheral markers of IP the role of zonulin has been recently recognized (18): this modulator of the tight junctions proved to be involved in autoimmune disorders typically associated with an IP dysfunction, such as celiac disease and type 1 diabetes (19). Notably, a recent work showed that zonulin can rapidly increase both IP permeability and blood brain barrier (BBB) *in vitro* by modifying TJs, having a synergic action with the pro-inflammatory cytokines typically involved in MS pathogenesis, and explaining, at least in part, how the gut-brain axis may mediate the pathogenesis of neuro-inflammatory diseases (20). Another peripheral biomarker recently used to quantify IPC in MS is the Intestinal Fatty Acid Binding Protein (IFABP). IFABP is a cytosolic protein exclusively expressed by enterocytes and rapidly released into blood circulation upon cell stress (21). Some data indicated that serum IFABP was higher in people with MS than in healthy controls (22), while others reported no differences between patients and controls (13); the discrepancy is possibly due to diverse analytical methods, suggesting that the approach still needs optimization. A consensus on the best method(s) to evaluate IPC is certainly desirable in this phase: it may help to replicate results in different conditions, and to understand whether specific or shared gut barrier changes characterize each immune-mediated disorder.

Recent works have tried to link IPC with risk factors known to be associated to MS, such as the epidemiological evidence of increased MS prevalence in countries at high latitudes, where the sunlight is limited and the populations tend to have vitamin D deficiency (23). A recent review suggests that vitamin D deficiency reduces intestinal calcium absorption and leads to gut stasis and subsequent IPC. This would allow gut microbiota to transfer more endotoxins into the blood and to stimulate the production of inflammatory cytokines within the CNS (24). Another interesting link seems to be that between gut barrier and BBB breakdown: in a work above reported (20), the authors demonstrated that, at least *in vitro*, increase of zonulin, as well as of interleukin 17 and interferon gamma, provoked similar effects of IPC and BBB leakage, suggesting plausible vicious circles between intestinal dysfunction and neuro-inflammation. These data are in accord with those of Fasano and coworkers who measured serum levels of zonulin in relapsing–remitting MS, and found increased concentrations in phase of disease activity, while patients in remission showed serum levels comparable to those of controls (18).

A crucial point regarding the contribution of IPC to MS pathophysiology is the low-grade microbial translocation to systemic circulation and eventually to brain (25, 26). Along this line, gastrointestinal disorders with intestinal barrier breakdown, such as celiac diseases and inflammatory bowel diseases, show evidence of CNS demyelination or overt co-morbidity with MS in a proportion of patients (27). However, several works showed that, also in the absence of gastrointestinal diseases, a low-grade translocation of bacteria or bacterial products from the intestines into the circulation is present in MS, and correlates with changes of gut microbiota (26). In this context a failure of the protective function that commensal bacteria exert on the gut barrier can be hypothesized. Homeostatic microbiota may regulate IP through multiple mechanisms: production of short chain fatty acids (SCFA) that increase tight junctions; toll like receptors activation, that promote epithelial cell proliferation, IgA synthesis, and antimicrobial peptides production; metabolizing actions on dietary tryptophan and production of metabolites that play a role as anti-inflammatory mediators also far from gut (28). Conversely, a low-grade endotoxemia, possibly due to IPC, was demonstrated in MS patients (29, 30). These works showed increase levels of lipopolysaccharide (LPS) and LPS-binding protein in plasma of MS patients, that correlated with the concentrations of pro-inflammatory cytokine and with the expanded disability status scale. In fact, LPS is known to exert pro-inflammatory actions on microglia and astrocytes, and to participate in the disruption of BBB, all effects that can perpetuate the pathogenic loop of MS. Along this line, a recent work showed that the circulating bacterial peptidoglycan comes from host microbiota and acts as a natural immune potentiator that tunes the host immune response. The same work also showed that the neutralization of the circulating peptidoglycan suppressed the development of the experimental model of MS (31).

A recent interesting observation concerning the gut-brain axis is the potential neuroactive impact of the human microbiota on mental status, in particular the quality of life and the depressive status. Clear metagenomic profiles and specific metabolites production (SCFA and neuromodulators' precursors) by gut prokaryotes have been reported to correlate with indicators of mental health, by surveying a large microbiome population cohort, with validation in independent data sets (32). These data bear relevance to the long known relationship between MS and affective disorders. Patients with MS have an estimated prevalence of depression that is 2–3 times higher than that of the general population (33). Mechanisms underlying this condition may be multiple: besides the reactive component due to the stressors of “living with MS,” lesion burden and brain atrophy are often correlated to standard scales for measuring the mood status. However, a new evidence (34) on a wide array of biological abnormalities shared by MS and major depressive disorder (peripheral inflammation, neuroinflammation, chronic oxidative and nitrosative stress, mitochondrial dysfunction, neuroendocrine abnormalities and microglial pathology) make it plausible that IPC, with gut dysbiosis and bacterial translocation into the systemic circulation, could represent a significant (albeit not the sole) determinant of mood status disruption in MS. This perspective may have therapeutic implications, suggesting new treatments to deal with depression in MS, that are closer to etiopathogenic rather than symptomatic approaches.

### Translational Implications

Among the translational implication of IPC, two points should be emphasized: the possible effects of disease-modifying therapies (DMT) on gut barrier and the potential therapeutic approaches aimed at antagonizing the IPC through stabilizers or enhancers of intestinal integrity. A recent review reported that DMTs, currently used in clinical practice for MS, would be able to act at different levels on IPC, modulating the gut barrier, the gut microbiota and the interaction between the two. However, these actions, though plausible, seem indirect, and whether they actually play a meaningful role in the clinical response remains to be established. Among these drugs, dimethyl-fumarate and fingolimod seem to have an antimicrobial action and a positive direct effect on TJ (26). On the other hand, teriflunomide and dimethyl-fumarate provoke gastro-intestinal side effects in some people with MS, raising the question whether these unwanted consequences may be associated, at least in part, to IP disruption. Actually, the effects of current DMT on IP was not yet explicitly evaluated; a study on dimethyl-fumarate on IP and microbiota was recently carried out in our Center (manuscript in preparation).

Besides being of pathophysiological interest, the brain-gut axis abnormalities (microbiota unbalance, IPC and alterations in bile acid metabolism) could also open new avenues for therapeutic targets. The probiotics use and the successful modification of the microbiome could be one strategy to modulate and improve intestinal barrier function. However, probiotics do not modify the host microbiome in a satisfying and lasting manner (few clinical trials in MS showed modest beneficial trends in clinical variables and some biomarker changes in peripheral immune function). Fecal microbiota transplantation would constitute the optimal strategy and isolated cases were described with beneficial effects on disease course. Also supplementation of bile acids might have several beneficial effects, modulating the intestinal barrier function, shaping the gut microbiota toward homeostatic profiles, and also regulating inflammatory signaling in the central nervous system (35). At least some of these effects may be mediated by the nuclear hormone receptor, farnesoid X receptor (FXR), that has bile acids among its ligands. Obeticholic acid (6α-ethyl-chenodeoxycholic acid), a synthetic FXR agonist, that is an orally available drug currently in clinical trials for the treatment of inflammatory diseases (alcoholic hepatitis, nonalcoholic steatohepatitis, and primary biliary cirrhosis), was shown to be capable of ameliorating EAE (36).

The topic of IP enhancers or stabilizers recently received increasing interest, being the object of several works: this approach seems to target an early pathogenic event (the IPC), underlying many conditions of chronic inflammation in gut and far-from-gut organs. Many drugs come from studies conducted in chronic gastro-enteric inflammation: an interesting example is larazotide (a 8-mer peptide with activity as TJ regulator), that was tested in a model of celiac disease and was shown to inhibit gliadin-induced macrophage accumulation in the intestine and to preserve the TJ structure (37). Larazotide was then tested in several trials in celiac disease with encouraging results (38–40). Another compound of interest is divertin, a small molecule that diverts myosin light chain kinase from its effects on gut barrier dysfunction and disease progression in experimental inflammatory bowel disease (41). Other approaches exploit the effects of probiotic or engineered bacteria to revert IPC and restore the gut barrier homeostasis. Elafin, an endogenously produced inhibitor of elastase that is deficient in inflammatory bowel diseases and gluten disorders, was effective in stabilizing IP and restoring gut homeostasis in a pilot study with food-grade bacteria engineered to produce the molecule (42, 43). In a study on EAE, where the authors confirm the pathogenic role of a profound defect in the IP function, treatment with oral daily probiotic *Escherichia coli* strain Nissle 1917 (ECN), but not with another strain, repaired intestinal permeability dysfunction and induced a general modulation of immune effectors, with a beneficial effect on the disease progression (44). Vitamin D, that is currently under active scrutiny in several chronic inflammatory conditions, including MS, has a specific action as a gut barrier stabilizer; a recent controlled trial in patients with inflammatory bowel disease reported improvement of IPC at different segments of gastro-intestinal tract (45). Finally, a recent paper clarifies the molecular model through which commensals act as enhancers of gut barrier integrity, disclosing possible therapeutic targets to counteract systemic inflammation. Sensing of commensal species by the C-type lectin receptor Mincle, coupled to a dendritic cell Syk-kinase, activates an homeostatic cascade that regulates the function of group 3 innate lymphoid cells, fosters the IgA production, and impedes the systemic translocation of gut microbiota (46).

All these findings provide several hints to try therapeutic approaches in MS: repurposing compounds that have been studied especially for IPC in chronic gastro-intestinal inflammation, and/or reworking the increasingly growing data coming from microbiota studies in EAE and MS will plausibly yield fruitful lines of attack against neuroinflammation.

## Author Contributions

All authors listed have made a substantial, direct and intellectual contribution to the work, and approved it for publication.

## Conflict of Interest Statement

The authors declare that the research was conducted in the absence of any commercial or financial relationships that could be construed as a potential conflict of interest.
